# Real‐world glycaemic outcomes observed with the use of Medtronic 780G, Tandem Control‐IQ and Omnipod 5 automated insulin delivery systems

**DOI:** 10.1111/dme.70251

**Published:** 2026-02-12

**Authors:** Muhammad S. Khan, Sathana Sivanantham, Lavanta Farouk, Deeksha Reddy Lakshmareddy, Sara De Scolasticis, Elisabeth Cresta, Ian Godsland, Parizad Avari, Nick Oliver, Lalantha Leelarathna, Monika Reddy

**Affiliations:** ^1^ Imperial College School of Medicine Imperial College London London UK; ^2^ Department of Metabolism, Digestion and Reproduction Imperial College London London UK; ^3^ Imperial College Healthcare NHS Trust London UK; ^4^ School of Medicine Keele University Keele Staffordshire UK

**Keywords:** continuous glucose monitoring, diabetes technology, hybrid automated insulin delivery, hybrid closed‐loop, type 1 diabetes

## Abstract

**Background:**

No randomised controlled trials have directly compared commercially available hybrid automated insulin delivery (AID) systems, and real‐world comparative data remain limited. This study evaluated glycaemic outcomes across three hybrid AID systems in adults with type 1 diabetes (T1D).

**Methods:**

This was a retrospective, observational, single‐centre study and included adults with T1D who transitioned from multiple daily injections (MDI) or non‐automated insulin pump therapy to hybrid AID. Data were collected from routinely used clinical data‐sharing platforms and electronic health records. Outcomes compared across systems included time in range (TIR; 3.9–10.0 mmol/L), time below range (TBR; <3.9 and <3.0 mmol/L), time above range (TAR; >10.0 and >13.9 mmol/L), glucose management indicator (GMI, %) and coefficient of variation (CV, %).

**Results:**

A total of 213 participants were included (Medtronic 780G *n* = 38; Tandem Control‐IQ *n* = 81; Omnipod 5 *n* = 94). After adjustment for baseline TIR, diabetes duration, insulin modality and AID use duration 780G users achieved a higher TIR increase (21.1% [95% CI 18.4–23.7]) compared to Control‐IQ (10.1% [3.2–17.3], *p* = 0.010) and Omnipod 5 (15.2% [12.9–17.5], *p* = 0.002), with corresponding reductions in TAR.

**Conclusion:**

All three hybrid AID systems were associated with improvements in glycaemic outcomes in real‐world use, supporting the role of AID systems in the management of T1D. Medtronic 780G use was associated with higher TIR increase compared with the other systems; however, these findings are based on measurements from different continuous glucose monitors between AID groups and cannot be used to infer superiority in glycaemic attainment.

**What's New?**

- Randomised controlled trials (RCTs) of automated insulin delivery (AID) systems have consistently demonstrated superior glycaemic outcomes compared with multiple daily insulin injections or conventional (non‐automated) insulin pump therapy. However, direct head‐to‐head RCTs comparing commercially available AID systems are lacking, and real‐world comparative data remain limited.
- In this real‐world analysis, we evaluated glycaemic outcomes with the use of Medtronic 780G, Tandem Control‐IQ and Omnipod 5. The Medtronic 780G was associated with a higher percentage of time in range (TIR) and reduced exposure to hyperglycaemia compared with the other two systems.
- These findings provide pragmatic, real‐world evidence to support clinical decision‐making and to assist individuals with type 1 diabetes in selecting among currently available AID systems.

## 1 INTRODUCTION

Automated insulin delivery (AID) systems facilitate optimal glucose levels in individuals with type 1 diabetes (T1D) by integrating insulin pumps with real‐time continuous glucose monitoring (CGM), while employing control algorithms to dynamically adjust insulin delivery rate based on sensor glucose levels.^1^ In the United Kingdom, the National Institute for Health and Care Excellence (NICE) recommends AID systems as a treatment option for managing T1D with specific access criteria.^2^ Following these recommendations (NICE TA943), AID uptake has increased substantially in the United Kingdom and is progressively becoming standard of care for T1D management.^3^

Currently, four commercially available AID systems are approved for use in the United Kingdom: Medtronic 780G, Tandem t:slim X2 with Control‐IQ (Tandem CIQ), Omnipod 5 (OP5) and CamAPS with YpsoPump or Dana pumps. These systems differ in multiple respects, including algorithm design, user‐adjustable parameters, interoperability with CGM devices, insulin reservoir capacity, pump configuration (tethered or tubeless) and clinical efficacy in improving the percentage of time spent in the target glycaemic range (TIR; 3.9–10 mmol/L).^4^, ^5^ These factors need to be considered when choosing the most appropriate AID for personalised self‐management of T1D.

All AID systems aim to optimise TIR and minimise hypoglycaemia. Medtronic 780G,^6^ Tandem CIQ^7^ and OP5^8^ have all shown reduced haemoglobin A1c (HbA1c) and increased TIR (%) in randomised controlled trials (RCTs) compared to Sensor‐Augmented Pump (SAP).

Despite robust evidence supporting the overall efficacy of AID systems, there are no head‐to‐head RCTs directly comparing their effectiveness. The few observational studies available have compared Medtronic 780G with Tandem CIQ,^9^, ^10^, ^11^, ^12^, ^13^ however, results are conflicting, with some studies reporting significant improvement in TIR with Medtronic 780G^9^, ^12^, ^13^ and others showing no difference between.^10^, ^11^ Notably, none of these studies were conducted in UK populations. Our study was conducted in a diverse UK population comparing these two hybrid AID systems, along with OP5, providing valuable real‐world evidence to guide clinical decision‐making.

We evaluated glucose outcomes across three hybrid AID systems, Medtronic 780G, Tandem Control‐IQ and Omnipod 5 in people with T1D. Participants were from diabetes clinics at a large NHS Trust providing specialist care to approximately 2000 adults living with T1D in a multi‐ethnic urban setting.

## 2 METHODS

### 2.1 Study design and setting

This was a retrospective, observational study based in diabetes secondary care clinics across Imperial College Healthcare NHS Trust (ICHNT) in London, UK. Adults (age >18 years) with T1D using either the Medtronic 780G, Tandem CIQ or OP5 systems were identified from the ICHNT pump database. Data from all individuals who had transitioned from multiple daily injections of insulin (MDI), standalone insulin pumps or SAPs with or without predictive low glucose suspend to one of the AID systems were included.

As a service evaluation of routinely collected data, this study did not require ethical approval.

### 2.2 Study procedures

Baseline demographic data including age, gender, body mass index (BMI), diabetes duration, CGM type and insulin delivery modality pre‐AID start were collected from electronic patient records. Prior insulin delivery method was classified as either MDI or pump. CGM type was also collected post‐AID. Sensor glucose data were collected from data‐sharing web‐based platforms used in routine clinical care (Dexcom Clarity, Abbott LibreView, Medtronic CareLink and Tidepool).

Baseline sensor data were collected from the 4‐week period immediately preceding AID initiation. If insufficient data or low sensor usage (<70%) made this timeframe unsuitable for analysis, an alternative 4‐week continuous period was selected. For this alternate pre‐AID data collection window, we selected a timeframe as close to the AID initiation date as feasible, and the maximum deviation was 3 months before AID start. Post‐AID data were collected from the latest 4 weeks of data available with >70% sensor usage, provided this occurred at least 3 months after AID initiation.

Glycaemic control outcomes assessed included TIR (%) (3.9–10 mmol/L), time below range (TBR <3.9, <3.0 mmol/L), time above range (TAR, >10.0, >13.9 mmol/L), glucose management indicator (GMI%) and coefficient of variation (%CV). These defined CGM metrics are recommended by international consensus guidelines.^14^ Outcomes were collected at baseline and post‐AID. The change from baseline to post‐AID in these outcomes was calculated with the primary outcome being the change in TIR.

To assess socio‐economic deprivation, postcode data were collected from electronic patient records, anonymised and linked to the Index of Multiple Deprivation (IMD).^15^ The IMD was derived from multiple domains, including income, employment, education, health and disability, barriers to housing and services and living environment. The deciles range from 1 to 10, where decile 1 represents the most deprived 10% of the UK population.

### 2.3 Statistical analyses

Data analysis was conducted using SPSS version 29 (IBM Corp, Armonk, NY). Data were assessed for normality using the Shapiro–Wilk test. Normally distributed variables were presented as mean and standard deviation (SD). For change in scores (i.e. post‐AID minus baseline) that were normally distributed, results are presented as mean and 95% confidence interval (CI). Variables with non‐normal distributions (including change variables) are reported as median and interquartile range (IQR).

Demographics and other baseline non‐glycaemic outcomes were compared across the AID groups using the Kruskal–Wallis test for continuous variables and chi‐squared tests for categorical variables. To account for baseline imbalances, variables showing significant between‐group differences were included as covariates in an analysis of covariance (ANCOVA) comparing changes in glycaemic outcomes among the three AID systems.

Between‐group comparisons of changes in glycaemic outcomes (post‐AID minus baseline) were performed using ANCOVA, with change in TIR (%) specified as the primary outcome. The ANCOVA models adjusted for the baseline value of the outcome (e.g. baseline TIR), prior insulin modality, diabetes duration and duration of AID use. If a change variable did not satisfy normality, a normal‐score transformation (Blom's formula) was applied prior to ANCOVA. If transformation failed to achieve normality, the Kruskal–Wallis test was used instead of ANCOVA. In the event of statistically significant differences, pairwise post‐hoc comparisons were conducted with Bonferroni correction for multiple testing.

We performed a sensitivity analysis to account for missing HbA1c data where we repeated the ANCOVA while only including people with an available baseline HbA1c so we could adjust for it. We also did a sensitivity analysis where we repeated the ANCOVA and excluded individuals with glucose data collected from alternate data collection periods for pre‐AID.

In the exploratory analyses, the influence of participant characteristics (age group, BMI, ethnicity, IMD and prior insulin modality) on change in TIR (%) was assessed using ANCOVA models adjusted for baseline TIR (%).

ANCOVA models were assessed for homogeneity of variance using Levene's test. Where Levene's test suggested violation of the homogeneity assumption (*p* < 0.05), we applied bootstrapping with 10,000 resamples using bias‐corrected and accelerated (BCa) confidence intervals to obtain robust estimates under heteroscedasticity. Model inferences were based on the bootstrapped estimates when assumption violations were present.

Within each AID group, comparisons between baseline and post‐AID values for glycaemic outcomes were made using paired *t*‐tests (for normally distributed variables) or Wilcoxon signed‐rank tests (for non‐normal variables).

All statistical tests were two‐tailed and a *p*‐value of <0.05 was considered statistically significant.

## 3 RESULTS

A total of 213 adults with T1D were enrolled in this study, of which 43.2% were men. Baseline demographics are summarised in Table 1. The combined median (interquartile range [IQR]) age of the participants was 39.0 years [29.0, 52.0], BMI 25.8 kg/m^2^ [23.1, 29.8], diabetes duration 21.0 years [12.0, 28.0] and baseline HbA1c 59 mmol/mol (7.5%) [53, 65]. A majority of the cohort, 69%, had transitioned from a pump and the total cohort spent a median time of 8.0 months [4.8, 22.0] on AID therapy. Exactly half of the participants were from a higher IMD (lower deprivation) (6–10), and 57.7% were of white ethnicity. When comparing baseline demographics across the three AID systems, there were significant differences in prior insulin management (*p* < 0.001) where the Tandem CIQ group had a much lower proportion of people who had switched from MDI as their previous management compared to the other two system groups. There were also significant baseline differences between groups in diabetes duration (*p* < 0.001), length of AID use (*p* < 0.001) and HbA1c (*p* = 0.018). Other demographics were not significantly different between the AID systems.

**TABLE 1:** Baseline group comparison between different automated insulin delivery systems.

Demographic variables	Combined (*n* = 213)	Medtronic 780G (*n* = 38)	Tandem control‐IQ (*n* = 81)	Omnipod 5 (*n* = 94)	*p* value*
Sex (%)	Male: 92 (43.2%) Female: 121 (56.8%)	Male: 18 (47.4%) Female: 21 (52.6%)	Male: 39 (48.1%) Female: 42 (51.9%)	Male: 35 (37.2%) Female: 59 (62.8%)	0.295
Age (years)	39.0 [29.0–52.0]	40.0 [31.8–56.0]	41.0 [28.0–52.5]	36.0 [27.8–51.5]	0.276
BMI (kg/m^2^)	25.8 [23.1, 29.8] (*n* = 185)	27.3 [23.0, 30.2] (*n* = 35)	26.6 [23.4, 31.5] (*n* = 70)	25.0 [23.0, 28.7] (*n* = 80)	0.125
IMD (%)	1–5: 100 (50.0%) 6–10: 100 (50.0%)	1–5: 20 (54.1%) 6–10: 17 (45.9%)	1–5: 35 (47.3%) 6–10: 39 (52.7%)	1–5: 45 (50.6%) 6–10: 44 (49.4%)	0.790
Prior insulin management (%)	MDI: 66 (31.0%) Pump: 147 (69.0%)	MDI: 12 (31.6%) Pump: 26 (68.4%)	MDI: 2 (2.5%) Pump: 79 (97.5%)	MDI: 52 (55.3%) Pump: 42 (44.7%)	**<0.001**
Diabetes duration (years)	21.0 [12.0, 28.0]	25.0 [18.0, 29.5]	23.0 [17.0, 31.5]	14.0 [8.8, 25.0]	**<0.001**
Duration of AID (months)	8.0 [4.8, 22.0]	13.0 [6.0, 21.3]	23.0 [12.0, 36.5]	4.9 [3.9, 6.5]	**<0.001**
Ethnicity (%)	White: 123 (57.7%) Other: 90 (42.3%)	White: 19 (50.0%) Other: 19 (50.0%)	White: 46 (56.8%) Other: 35 (43.2%)	White: 58 (61.7%) Other: 36 (38.3%)	0.457
Baseline HbA1c (mmol/mol)	59.0 [53.0, 65.0] (*n* = 177)	61.0 [55.8, 66.0] (*n* = 30)	55.0 [49.5, 61.0] (*n* = 53)	59.0 [53.0, 67.0] (*n* = 94)	**0.018**
Baseline HbA1c (%)	7.5%	7.7%	7.2%	7.5%	

*Note*: Data presented as median [IQR] or *n* (%). Continuous variables with missing data, number of participants (*n*) is given. *p* values of < 0.05 are significant and highlighted in bold.

Abbreviations: AID, Automated Insulin Delivery; BMI, body mass index; IMD, index of multiple deprivation; MDI, multiple daily injections.

* Chi‐squared test was used for categorical variables, and the Kruskal–Wallis test was used for continuous variables.

After comparing glucose outcomes pre‐AID to post‐AID for the three AID systems, all three AID systems showed significant improvements in: TIR (%) (all *p* < 0.001), TBR <3.9 mmol/L (%) (780G: *p* < 0.001, CIQ: *p* = 0.008, OP5: *p* < 0.001), TAR >10.0 mmol/L (%) (all *p* < 0.001), TAR >13.9 mmol/L (%) (all *p* < 0.001), GMI (%) (780G: *p* < 0.001, CIQ: *p* = 0.004, OP5: *p* < 0.001) and CV (%) (780G: *p* < 0.001. CIQ: *p* < 0.001, OP5: *p* = 0.001) (Table 2).

**TABLE 2:** Glucose outcomes before and after transitioning to an automated insulin delivery system.

Glucose outcomes	Baseline	Post‐AID	*p* value*
Medtronic 780G (*n* = 38)			
Time in range (%) (3.9–10.0 mmol/L)^a^	52.0 (±14.2)	73.4 (±11.0)	**<0.001**
Time below range (%) <3.9 mmol/L	2.0 [0.0, 4.0]	1.0 [0.0, 2.0]	**<0.001**
Time below range (%) <3.0 mmol/L	0.0 [0.0, 0.3]	0.0 [0.0, 0.0]	0.058
Time above range (%) >10 mmol/L	49.0 [34.5, 53.3]	25.5 [19.0, 29.3]	**<0.001**
Time above range (%) >13.9 mmol/L	17.0 [9.8, 22.0]	5.0 [2.0, 7.0]	**<0.001**
Glucose management indicator (%)	7.7 [7.2, 8.0]	6.9 [6.7, 7.1]	**<0.001**
Coefficient of variation (%)	36.8 [33.4, 40.8]	32.5 [29.4, 35.2]	**<0.001**
Tandem Control‐IQ (*n* = 81)			
Time in range (%) (3.9–10.0 mmol/L)^a^	59.3 (±16.1)	67.0 (±14.4)	**<0.001**
Time below range (%) <3.9 mmol/L	1.0 [1.0, 3.0]	1.0 [0.0, 2.0]	**0.008**
Time below range (%) <3.0 mmol/L	0.0 [0.0, 0.0]	0.0 [0.0, 0.0]	0.264
Time above range (%) >10 mmol/L	36.5 [24.0, 50.3]	31.0 [18.0, 44.0]	**<0.001**
Time above range (%) >13.9 mmol/L	9.0 [4.8, 19.5]	7.5 [2.0, 13.3]	**<0.001**
Glucose management indicator (%)	7.3 [6.8, 7.8]	7.1 [6.8, 7.6]	**0.004**
Coefficient of variation (%)	35.5 [32.6, 4.1]	34.7 [30.5, 38.2]	**0.001**
Omnipod 5 (*n* = 94)			
Time in range (%) (3.9–10.0 mmol/L)^a^	51.9 (±16.5)	67.5 (±11.4)	**<0.001**
Time below range (%) <3.9 mmol/L	1.5 [1.0, 3.0]	1.0 [0.0, 2.0]	**<0.001**
Time below range (%) <3.0 mmol/L	0.0 [0.0, 0.0]	0.0 [0.0, 0.0]	**0.020**
Time above range (%) >10 mmol/L	44.0 [32.8, 57.0]	30.5[23.8, 40.0]	**<0.001**
Time above range (%) >13.9 mmol/L	15.0 [8.0, 26.0]	7.0 [4.0, 13.0]	**<0.001**
Glucose management indicator (%)	7.6 [7.1, 8.1]	7.1 [6.9, 7.5]	**<0.001**
Coefficient of variation (%)	36.6 [33.2, 39.7]	34.1 [31.2, 36.5]	**<0.001**

*Note*: *p* values of < 0.05 are significant and highlighted in bold.

^a^ Normally distributed variable presented as mean (±SD). 95% Remaining data presented as median [IQR].

* *p*‐values were obtained via paired *t*‐tests for normally distributed variables and non‐parametric Wilcoxon matched‐pair signed‐rank tests for non‐normally distributed variables.

When comparing adjusted change (post‐AID–pre‐AID) in glucose outcomes between the three AID groups, there were significant differences between groups in TIR (%) (*p* < 0.001), TAR >10.0 mmol/L (%) (*p* = 0.004), TAR >13.9 mmol/L (%) (*p* < 0.001), GMI (%) (*p* < 0.001) and CV (%) (*p* = 0.041) (Table 3).

**TABLE 3:** Comparison of change in glucose outcomes between the three automated insulin delivery systems.

Glucose outcomes	Medtronic 780G (*n* = 38)	Tandem control‐IQ (*n* = 81)	Omnipod 5 (*n* = 94)	*p* value*
Change in time in range (%) (3.9–10.0 mmol/L)^a^	+21.5 (+18.0, +24.9)	+7.7 (+5.1, +10.3)	+15.6 (+13.1, +18.1)	**<0.001**
Change in time in range (%) (3.9–10.0 mmol/L)^b^, ^e^ (adjusted)	+21.1 (+18.3, +23.7)	+10.1 (+3.2, +17.3)	+15.2 (+12.9, +17.5)	**<0.001**
Change in time below range (%) <3.9 mmol/L^c^	−1.0 [−2.3, 0.0]	0.0 [−1.0, 0.0]	−1.0 [−2.0, 0.0]	0.483
Change in time below range (%) <3.0 mmol/L^d^	0.0 [0.0, 0.0]	0.0 [0.0, 0.0]	0.0 [0.0, 0.0]	0.599
Change in time above range (%) >10 mmol/L^c^, ^e^	−19.0 [−28.3, −12.8]	−6.0 [−13.0, +2.3]	−12.5 [−24.3, −4.0]	**0.004**
Change in time above range (%) >13.9 mmol/L^c^, ^e^	−12.0 [−19.3, −4.8]	−2.5 [−6.0, +2.0]	−5.0 [−15.3, −1.0]	**<0.001**
Change in glucose management Indicator (%)^c^, ^e^	−0.7 [−1.1, −0.4]	−0.1 [−0.4, +0.1]	−0.4 [−0.9, 0.0]	**<0.001**
Change in coefficient of variation (%)^c^	−4.9 [−9.4, −1.8]	−1.6 [−5.2, +1.5]	−2.6 [−5.9, +0.2]	**0.041**

*Note*: *p* values of < 0.05 are significant and highlighted in bold.

Abbreviations: AID, automated insulin delivery; ANCOVA, analysis of covariance.

^a^ Normally distributed and displayed as original value, mean (95% CI). *p*‐value obtained from ANCOVA with no covariates adjusted for.

^b^ Normally distributed and adjusted value accounting for all covariates*, mean (95% CI).

^c^ Transformed for analysis. Displayed as original (median [IQR]).

^d^ Untransformed for analysis. Presented as median [IQR].

^e^ Violated homogeneity of variance. Analysed using Bias‐corrected and accelerated CI bootstrapped ANCOVA (10,000 samples).

* *p*‐values for normally distributed adjusted (and transformed) variables were obtained from ANCOVA, adjusting for baseline value of the tested variable, previous insulin modality, diabetes duration and AID duration. *p*‐values for non‐transformed variables were obtained with Kruskal–Wallis test. *Post hoc* tests were applied with the Bonferroni correction.

**FIGURE 1:**
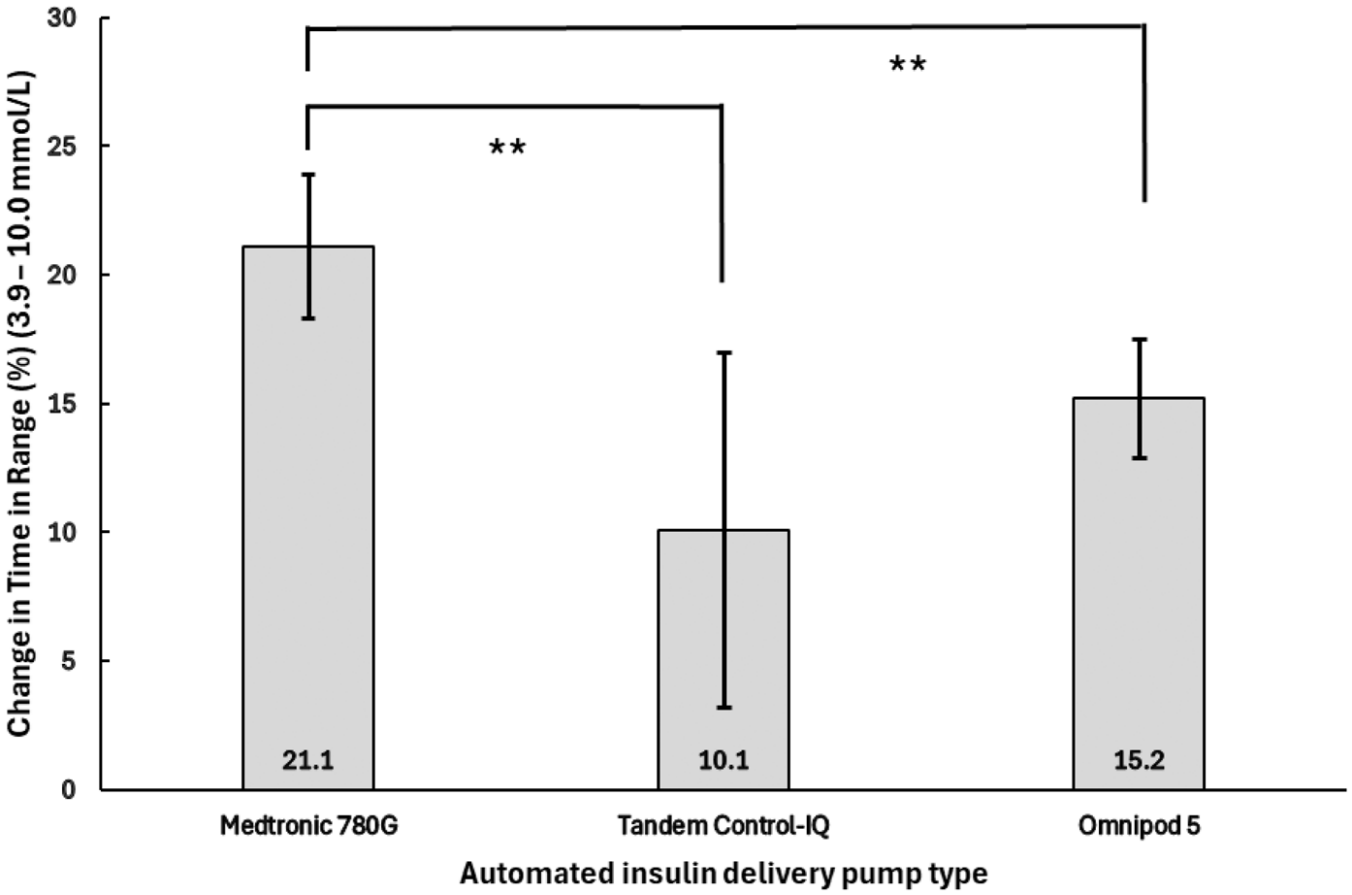
Adjusted change in time in range (%) (3.9–10 mmol/L) compared across automated insulin delivery systems. *p < 0.05, **p < 0.01, ***p < 0.001. ANOVA adjusted for baseline time in range (%), previous insulin modality, diabetes duration and automated insulin delivery duration. Analysed using bias‐corrected and accelerated CI bootstrapped ANCOVA (10,000 samples) due to violation of homogeneity of variance. Data presented as mean (95% CI).

Medtronic 780G users demonstrated the largest improvement in mean [95%CI] TIR (%), with an increase of 21.1% [18.3, 23.7]. *Post hoc* analysis with pairwise comparisons confirmed this increase was significantly greater than that observed with CIQ (TIR: 10.1% [3.2, 17.3], *p* = 0.010) and OP5 (TIR: 15.2% [12.9, 17.5], *p* = 0.002) (Table 3 and Figure 1).

Users of 780G demonstrated significantly larger reductions in median [IQR] TAR >10 mmol/L (%) (−19.0% [−28.3, −12.8]) compared with CIQ (−6.0% [−13.0, 2.3], *p* = 0.009) and OP5 (−12.5% [−24.3, −4.0], *p* = 0.002). Similarly, the reduction in median [IQR] TAR >13.9 mmol/L (%) with 780G (−12.0% [−19.3, −4.8]) was greater than with CIQ (−2.5% [−6.0, 2.0], *p* = 0.016) and OP5 (−5.0% [−15.3, −1.0], *p* < 0.001).

The 780G group also showed the largest decrease in median [IQR] GMI (%), with a reduction of −0.7% [−1.1, −0.4], which was greater than that with CIQ (−0.1% [−0.4, 0.1], *p* = 0.002) and OP5 (−0.4% [−0.9, 0.0], *p* < 0.001). Similarly, CV (%) improved significantly in 780G users with a median [IQR] reduction of −4.9% [−9.4, −1.8]. This reduction was significantly greater than that observed with CIQ (−1.6% [−5.2, 1.5], *p* = 0.041), but not significantly greater than OP5 (−2.6% [−5.9, 0.2], *p* = 0.598).

There were no significant differences between the systems in the change in TBR <3.9 (%) or TBR <3.0 mmol/L (%). No significant differences were observed between CIQ and OP5 for any glucose outcome.

The sensitivity analysis to account for missing HbA1c data (Table S2) did not alter the significant findings across the AID systems. However, the sensitivity analysis which excluded those with glucose data collected from alternate data collection periods for pre‐AID (Table S3) showed that there was no longer any significant difference between AID groups in CV (%). Other significant findings between groups remained consistent.

Baseline CGM subtype differed between AID groups, with more 780G users using Freestyle Libre sensors and more CIQ/OP5 users using Dexcom sensors. Post‐AID sensors aligned with device compatibility (780G—Guardian 4/Simplera Sync, CIQ—Dexcom G6/G7, OP5—Dexcom G6/Freestyle Libre 2+, Table S4).

## 4 DISCUSSION

This retrospective observational study demonstrated that the Medtronic 780G, OP5 and Tandem CIQ AID systems all led to significant improvements in glucose metrics. Among these, the Medtronic 780G system achieved greater improvements in percentage time in range and reductions in hyperglycaemia, with comparable hypoglycaemia rates relative to the other two systems.

Consistent with findings from previous RCTs of each individual system^6^, ^7^, ^8^ our data confirm that AID systems provide benefits over standard care (Table 2). This reinforces that all three hybrid systems, when appropriately initiated and supported, are effective options for self‐management of T1D.

There remains a paucity of direct comparative evidence between commercial AID systems, with no head‐to‐head RCTs and limited observational studies. Bassi et al.^9^ performed an observational study in Italy, comparing Medtronic 780G to Tandem CIQ and reported a greater TIR (%) improvement in Medtronic 780G users compared to Tandem Control‐IQ users (+19.1% vs. +9.8%), aligning closely with our findings (+21.1% vs. +10.1%, *p* = 0.010, Table 3). Two additional studies^12^, ^13^ reported similar findings, whereas others^10^, ^11^ found no difference. These conflicting results likely reflect differences in design and patient selection. A previous study^10^ included only 6‐ to 18‐year‐olds transitioning from predictive low glucose suspend plus SAP, unlike our cohort which comprised adults with differing prior therapies. Moreover, the prospective nature of previous studies^10^, ^11^ may have introduced behavioural bias through increased engagement and monitoring (Hawthorne effect), while our retrospective approach captured outcomes reflective of routine clinical practice.

Apart from TIR (%), Medtronic 780G caused significantly greater improvement in TAR >10 mmol/L (%) (*p* = 0.009), TAR >13.9 mmol/L (%) (*p* = 0.016), GMI (%) (*p* = 0.002) and CV (%) (*p* = 0.041) compared to Tandem CIQ. Against OP5, Medtronic 780G caused significantly greater improvement in TIR (%) (*p* = 0.002), TAR >10 mmol/L (%) (*p* = 0.002), TAR >13.9 mmol/L (%) (*p* < 0.001) and GMI (%) (*p* < 0.001). To our knowledge, this is the first observational study to compare Medtronic 780G and OP5. Therefore, our findings provide novel real‐world observations that 780G may be associated with greater increases in TIR (%) while maintaining equivalent TBR (%) compared to the other two systems.

By contrast, Tandem CIQ and OP5 performed similarly across all glucose metrics. This is consistent with findings from Gera et al.,^16^ who found no significant differences in TIR (%) improvement, suggesting comparable effectiveness between the two systems.

The reasons behind the greater TIR (%) increase observed with Medtronic 780G in this study remain uncertain. One potential factor could be differences in how frequently the hybrid AID systems deliver automated correction doses, with some systems allowing more frequent corrections than others.^17^ Additionally, variations in the target glucose levels set by different AID systems may contribute to differences in glucose outcomes. A more proactive approach in glucose targeting might enable more responsive control, and further understanding of these mechanisms could help guide the development and optimisation of future AID technologies.

As an exploratory analysis, we were interested to see if any demographic or other factors could impact the effectiveness of AID systems (Table S1). We observed that those who switched from MDI achieved greater significant improvement in TIR (%) compared to those who switched to pumps. Therefore, we adjusted for prior insulin modality in our comparisons of glucose outcomes between systems. One of the key strengths of this study is that it is one of the first to compare glycaemic outcomes across three widely used AID systems in the United Kingdom under NHS conditions. Unlike RCTs, which often exclude comorbidities and involve highly engaged participants with intensive follow‐up, our observational design reflects routine clinical practice. This real‐world analysis therefore provides evidence that is more representative of the broader T1D population managed within the NHS.

Another important strength of our study is the large sample size, which includes a high proportion of individuals from non‐white ethnic backgrounds (Table 1). Diverse cohorts remain uncommon in evaluations of AID systems, thereby enhancing the generalisability and clinical relevance of our findings.

Adjusting for covariates, including baseline glucose outcomes and demographics, also strengthens the internal validity of this study and reduces potential confounding.

One covariate we could not adjust for in our comparison of changes in glycaemic outcomes between AID systems was HbA1c. HbA1c was significantly different at baseline (Table 1) but could not be included as a covariate due to missing baseline HbA1c values. However, sensitivity analyses were performed where we adjusted for HbA1c as an additional covariate by only including people with baseline HbA1c values in the analysis (Table S2). This yielded similar results to the original analysis, improving the robustness of our results.

To ensure that the alternative data collection timeframes specified in our methodology for pre‐AID did not significantly impact our results, we conducted a sensitivity analysis excluding any data collected from these alternative timeframes (Table S3). We found that results for change in TIR (%) were still significantly different between AID systems, showing consistency of the results for TIR (%).

This study has several limitations. Given the retrospective nature of the study, we acknowledge the potential selection bias related to clinicians' preferred AID systems, along with missing data and the lack of protocolised follow‐up. As a non‐randomised observational study, residual confounding due to significant baseline imbalances cannot be excluded despite statistical adjustment. Thus, causal inference cannot be established. Moreover, heteroscedasticity was present in some of our ANCOVA models and is common in real‐world datasets with baseline imbalances. While robust bootstrapping was applied to mitigate this, some imprecision may persist and should be considered when interpreting system‐level differences. Although the 780G group had a sample size substantial for a real‐world retrospective cohort, compared to the other AID systems included, it was smaller, which may impact the precision of system comparisons. Differences in CGM sensors across groups post‐AID, necessitated by device incompatibility, and pre‐AID represents a potential source of bias in CGM‐derived outcomes (Supplementary Table 4).^18^ This lack of CGM consistency represents a key limitation and precludes confident inference of superiority between systems based on CGM‐derived outcomes.

Additionally, some individuals may not have received optimised treatment to get the best outcome due to suboptimal device settings or limited user training. Safety outcomes, user behaviour information (such as levels of user‐driven insulin delivery) and user device settings (such as glucose targets) were not collected in this study. These factors may have influenced glycaemic outcomes and represent a limitation of our analysis. As these data were not available in the present study, future studies should prospectively collect safety outcomes, user behaviour information and device‐specific system settings to better contextualise differences in glycaemic outcomes.^19^

Although the single‐centre nature of our cohort may limit the generalisability of our findings, it also eliminates inter‐site variability. All participants were managed by the same multidisciplinary team, ensuring consistency in clinical care and optimisation.

## 5 CONCLUSION

In summary, this real‐world study demonstrates that all three‐hybrid AID systems evaluated (Medtronic 780G, Tandem Control‐IQ and Omnipod 5) are associated with significant improvements in glycaemic control among adults living with T1D. Medtronic 780G users showed a greater increase in TIR (%) and reductions in hyperglycaemia; however, these comparisons are based on readings from different CGMs and cannot be used to infer superiority in glycaemic attainment. Overall, these findings support the beneficial role of hybrid AID systems in routine clinical care and their broader implementation within healthcare systems.

## FUNDING STATEMENT

The authors received no specific funding for this work.

## CONFLICT OF INTEREST STATEMENT

LL reports having received speaker honoraria from Abbott Diabetes Care, Insulet, Medtronic, Novo Nordisk, Roche and Sanofi; was on advisory panels for Abbott, Novo Nordisk, Dexcom, Medtronic, Sanofi and Roche; and received research support from Novo Nordisk, Abbott Diabetes Care and Dexcom. MR has received speaker honoraria from Dexcom; participated in advisory boards for Medtronic and Roche; and received research support from Medtronic, Roche and Dexcom. NO has received research support from Dexcom, Medtronic and Roche Diabetes; has participated in advisory groups for Dexcom, Medtronic and Roche Diabetes; and has received fees for speaking from Astra Zeneca, Sanofi, Dexcom, Tandem, Medtronic and Roche Diabetes. PA has received equipment from Dexcom for investigator‐initiated studies.

## Supporting information


Data S1.

## Data Availability

The data that support the findings of this study are available from Imperial College Healthcare NHS Trust. Restrictions apply to the availability of these data, which were used under license for this study. Data are available from the author(s) with the permission of Imperial College Healthcare NHS Trust.
