# Supplementary material for: Real‐world glycaemic outcomes observed with the use of Medtronic 780G, Tandem Control‐IQ and Omnipod 5 automated insulin delivery systems
Source: Diabet Med. 2026 Feb 12;43(5):e70251. doi: 10.1111/dme.70251 (PMC13074138; doi:10.1111/dme.70251)
Supplement: Supplementary file 1 — Data S1. [file DME-43-e70251-s001.docx]

**Supplementary Tables**

| **Supplementary table 1 - The impact of clinical characteristics and baseline demographics on the change in Time in Range (%) (3.9 to 10.0 mmol/l) across automated insulin delivery groups combined.** | | | |
| --- | --- | --- | --- |
| **Group** | **Baseline %Time in Range**  **Mean (SD)** | **Change in %Time in Range^a^**  **Mean (95% CI)** | **p-value*** |
| **Age groups (years)**^✝^ | | | |
| 18-35 (n=88) | 55.6 ± 16.4 | 12.5 (10.4, 14.5) | 0.147 |
| 36-49 (n=61) | 53.5 ± 16.6 | 15.6 (13.2, 18.1) |  |
| ≥50 (n=64) | 54.8 ± 16.1 | 13.3 (10.9, 15.7) |  |
| **BMI** | | | |
| ≤24.9 (n=82) | 53.1 ± 17.1 | 15.0 (12.8, 17.2) | 0.843 |
| 25-29.9 (n=60) | 54.8 ± 15.8 | 14.5 (12.0, 17.9) |  |
| ≥30 (n=43) | 52.7 ± 14.9 | 13.9 (10.8, 16.9) |  |
| **IMD** | | | |
| 1-5 (n=100) | 55.8 ± 16.4 | 14.1 (12.2, 16.1) | 0.562 |
| 6-10 (n=100) | 53.6 ± 16.3 | 13.3 (11.3, 15.3) |  |
| **Ethnicity** | | | |
| White (n=123) | 53.0 ± 15.9 | 13.5 (11.7, 15.2) | 0.768 |
| Not white (n=90) | 57.0 ± 16.7 | 13.9 (11.8, 15.9) |  |
| **Previous insulin modality**^✝^ | | | |
| MDI (n=66) | 54.1 ± 17.0 | 17.8 (15.5, 20.1) | <0.001 |
| Pumps (n=147) | 55.0 ± 16.0 | 11.8 (10.2, 13.3) |  |
| TIR – time in range, BMI – body mass index, IMD – index of multiple deprivation, MDI – multiple daily injections, ANCOVA – analysis of covariance  ^a^ Change in %Time in Range adjusted for baseline % Time in Range  ^✝^ Violated homogeneity of variance. Analysed using Bias-corrected and accelerated CI bootstrapped ANCOVA (10,000 samples).    *p-values were obtained via ANCOVA and post-hoc pairwise comparisons using the Bonferroni correction were performed if overall effects were significant. | | | |

| **Supplementary Table 2 – Change in glucose outcome analysis across automated insulin delivery systems while adjusting for HbA1c in those who had baseline HbA1c values** | | | | |
| --- | --- | --- | --- | --- |
| **Glucose outcomes** | **Medtronic 780G**  **(n=30)** | **Tandem Control-IQ**  **(n=53)** | **Omnipod 5**  **(n=94)** | **p-value** |
| Change in % Time in Range (3.9 – 10.0 mmol/L)^a✝^ | +22.8  (+19.9, +25.5) | +9.9  (+2.2, +18.1) | +15.3  (+12.9, +17.5) | **<0.001** |
| Change in % Time Below Range <3.9 mmol/L^b^ | -0.5  [-2.0, 0.0] | 0.0  [-1.0, 0.0] | -1.0  [-2.0, 0.0] | 0.838 |
| Change in % Time Below Range <3.0 mmol/L^c^ | 0.0  [0.0, 0.0] | 0.0  [0.0, 0.0] | 0.0  [0.0, 0.0] | 0.683 |
| Change in % Time Above Range >10 mmol/L^b✝^ | -19.5  [-29.3, -13.0] | -5.0  [-13.0, +3.0] | -12.5  [-24.3, -4.0] | **<0.001** |
| Change in % Time Above Range >13.9 mmol/L^b✝^ | -13.0  [-20.3, -6.5] | -2.0  [-8.5, +2.0] | -5.0  [-15.3, -1.0] | **<0.001** |
| Change in Glucose Management Indicator %^b✝^ | -0.8  [-1.1, -0.4] | -0.1  [-0.5, +0.1] | -0.4  [-0.9, 0.0] | **<0.001** |
| Change in % Coefficient of Variation^b^ | -4.9  [-9.4, -1.6] | -1.8  [-5.0, +1.6] | -2.6  [-5.9, +0.2] | **0.010** |
| ANCOVA – analysis of covariance, AID – Automated Insulin Delivery  **^a^** Normally distributed and adjusted value accounting for all covariates*, mean (95% CI).  **^b^** Transformed for analysis. Displayed as original (median [IQR]).  **^c^** Untransformed for analysis. Presented as median [IQR]  **^✝^** Violated homogeneity of variance. Analysed using Bias-corrected and accelerated CI bootstrapped ANCOVA (10,000 samples).  *p-values for normally distributed (and transformed) variables were obtained from ANCOVA, adjusted for baseline value of the tested variable, previous insulin modality, diabetes duration, baseline HbA1c and AID duration. Only included those with baseline HbA1c values. p-values for non-transformed variables were obtained with Kruskal-Wallis test. Post-hoc tests were applied with the Bonferroni correction. | | | | |

| **Supplementary Table 3 – Change in glucose outcome analysis across automated insulin delivery (AID) systems while excluding glucose data collected from alternate collection windows for pre-AID data** | | | | |
| --- | --- | --- | --- | --- |
| **Glucose outcomes** | **Medtronic 780G**  **(n=35)** | **Tandem Control-IQ**  **(n=73)** | **Omnipod 5**  **(n=90)** | **p-value*** |
| Change in Time in Range (%) (3.9 – 10.0 mmol/L)^a✝^ | +21.0  (+18.1, +23.9) | +10.0  (+3.1, +17.3) | +15.3  (+12.7, +17.7) | **0.002** |
| Change in % Time Below Range <3.9 mmol/L^b^ | -1.0  [-2.0, 0.0] | 0.0  [-1.0, 0.0] | -0.5  [-2.0, 0.0] | 0.637 |
| Change in % Time Below Range <3.0 mmol/L^c^ | 0.0  [0.0, 0.0] | 0.0  [0.0, 0.0] | 0.0  [0.0, 0.0] | 0.738 |
| Change in % Time Above Range >10 mmol/L^b✝^ | -20.0  [-29.0, -13.0] | -7.0  [-13.0, +2.5] | -12.5  [-24.3, -4.0] | **0.006** |
| Change in % Time Above Range >13.9 mmol/L^b✝^ | -12.0  [-20.0, -5.0] | -3.0  [-6.5, +1.5] | -5.0  [-15.3, -1.0] | **<0.001** |
| Change in Glucose Management Indicator %^b✝^ | -0.7  [-1.1, -0.4] | -0.2  [-0.5, +0.1] | -0.4  [-0.9, 0.0] | **<0.001** |
| Change in % Coefficient of Variation^b^ | -4.9  [-9.3, -1.7] | -1.4  [-5.0, +1.5] | -2.6  [-5.9, +0.3] | 0.082 |
| ANCOVA – analysis of covariance, AID – Automated Insulin Delivery  **^a^** Normally distributed and adjusted value accounting for all covariates*, mean (95% CI).  **^b^** Transformed for analysis. Displayed as original (median [IQR]).  **^c^** Untransformed for analysis. Presented as median [IQR]  **^✝^** Violated homogeneity of variance. Analysed using Bias-corrected and accelerated CI bootstrapped ANCOVA (10,000 samples).  *p-values for normally distributed (and transformed) variables were obtained from ANCOVA, adjusted for baseline value of the tested variable, previous insulin modality, diabetes duration, and AID duration. p-values for non-transformed variables were obtained with Kruskal-Wallis test. Post-hoc tests were applied with the Bonferroni correction. | | | | |

| **Supplementary Table 4 – Continuous glucose monitor (CGM) types used pre and post automated insulin delivery (AID) system use** | | | |
| --- | --- | --- | --- |
| **CGM types (%)** | **Medtronic 780G**  **(n=38)*** | **Tandem Control-IQ**  **(n=81)** | **Omnipod 5**  **(n=94)** |
| **Pre-AID** | | | |
| Freestyle Libre 2 / 2+ / 3 | 31 (81.6%) | 10 (12.3%) | 37 (39.4%) |
| Dexcom G6 | 6 (15.8%) | 68 (84.0%) | 55 (58.5%) |
| Dexcom G7 | 1 (2.6%) | 3 (3.7%) | 2 (2.1%) |
| **Post-AID** | | | |
| Freestyle Libre 2+ | 0 (0.0%) | 0 (0.0%) | 15 (16.0%) |
| Dexcom G6 | 0 (0.0%) | 58 (71.6%) | 79 (84.0%) |
| Dexcom G7 | 0 (0.0%) | 23 (28.4%) | 0 (0.0%) |
| Guardian 4 | 33 (86.8%) | 0 (0.0%) | 0 (0.0%) |
| Simplera Sync | 5 (13.2%) | 0 (0.0%) | 0 (0.0%) |
| AID – Automated insulin delivery, CGM – Continuous glucose monitor | | | |
